# Cycling Quiescence in Temozolomide Resistant Glioblastoma Cells Is Partly Explained by microRNA-93 and -193-Mediated Decrease of Cyclin D

**DOI:** 10.3389/fphar.2019.00134

**Published:** 2019-02-22

**Authors:** Jessian L. Munoz, Nykia D. Walker, Satvik Mareedu, Sri Harika Pamarthi, Garima Sinha, Steven J. Greco, Pranela Rameshwar

**Affiliations:** ^1^Rutgers New Jersey Medical School, Rutgers University, Newark, NJ, United States; ^2^Rutgers School of Graduate Studies at New Jersey Medical School, Rutgers University, Newark, NJ, United States

**Keywords:** glioblastoma, cell cycle, chemoresistance, microRNA, Cyclin D

## Abstract

Glioblastoma multiforme (GBM) is a fatal malignancy of the central nervous system, commonly associated with chemoresistance. The alkylating agent Temozolomide (TMZ) is the front-line chemotherapeutic agent and has undergone intense studies on resistance. These studies reported on mismatch repair gene upregulation, ABC-targeted drug efflux, and cell cycle alterations. The mechanism by which TMZ induces cell cycle arrest has not been well-established. TMZ-resistant GBM cells have been linked to microRNA (miRNA) and exosomes. A cell cycle miRNA array identified distinct miRNAs only in exosomes from TMZ-resistant GBM cell lines and primary spheres. We narrowed the miRs to miR-93 and -193 and showed in computational analyses that they could target Cyclin D1. Since Cyclin D1 is a major regulator of cell cycle progression, we performed cause-effect studies and showed a blunting effects of miR-93 and -193 in Cyclin D1 expression. These two miRs also decreased cell cycling quiescence and induced resistance to TMZ. Taken together, our data provide a mechanism by which GBM cells can exhibit TMZ-induced resistance through miRNA targeting of Cyclin D1. The data provide a number of therapeutic approaches to reverse chemoresistance at the miRNA, exosomal and cell cycle points.

## Introduction

Glioblastoma multiforme (GBM) is the most common adult primary brain tumor. In addition, GBM is also the most lethal brain cancer with a 5-year survival rate of ∼8% (Naydenov et al., 2011). GBMs uniformly acquire resistance to the front-line alkylating chemotherapeutic agent, Temozolomide (TMZ) (Jiang et al., 2011). Multiple mechanisms of TMZ resistance have been previously described such as an increase in the Multiple Drug Resistance (MDR) efflux, base pair excision, gene methylation and cell cycle arrest (Johannessen and Bjerkvig, 2012).

Cell cycle progression requires the concerted activity of a number of factors both positive and negative (Louis, 2006). Cyclin D1 is a G1 phase protein that facilitates the progression of cells into S phase. The expression and degradation of Cyclin D1 is highly regulated to coordinate the temporal role of Cyclin D1 (Freemantle et al., 2007). Since microRNAs (miRs) have been linked to chemoresistance, we studied if miRNAs, through the regulation of Cyclin D1, affected the response of GBM cells to TMZ.

miRNA are small RNA molecules (18–22 base pairs) that regulate protein expression by binding to the non-coding regions of mRNA to inhibit translation (Sen et al., 2014). Increasing evidence has shown roles for miRNAs in development, differentiation and malignancy (Di Leva et al., 2014). In addition, miRNAs have been shown to have a key role in the acquisition and maintenance of chemoresistance (Tezcan et al., 2014). miRNAs can also target multiple regulators of cell cycle progression including Cyclin D1 (Zhao et al., 2014; Huang et al., 2015).

Exosomes are endosomal in origin and can function as a mediator of contact-independent communication among cells (Pant et al., 2012). The exosomes contain RNA including non-coding miRNA, DNA and proteins (Thery et al., 2002). Due to their ability to mediate intercellular communication, exosomes could also serve as vehicles for miRNA-based therapy (Munoz et al., 2013). An understanding of how miRNAs affect GBM pathophysiology will offer opportunities for novel therapeutic approaches. This study shows describes roles for two miRNAs, miR-93 and -193, in regulating Cyclin D1 expression. We also demonstrated that the regulation of Cyclin D1 by these two miRNAs led to TMZ resistance in GBM cells.

## Materials and Methods

### Cell Lines

U87 and T98G cells were purchased from American Type Culture Collection (ATCC; Manassas, VA) and then expanded as per manufacturer’s instructions.

### Reagents and Antibodies

All tissue culture media were purchased Life Technologies Gibco (Grand Island, NY), fetal calf serum (FCS) from Hyclone Laboratories (Logan, UT), TMZ from Sigma Aldrich (St. Louis, MO) and, anti-Cyclin D1, anti-β-actin, HRP-anti-rabbit and HRP-anti-mouse IgG from Cell Signaling (Danvers, MA).

### Real-Time RT-PCR

RNA was extracted with Trizol reagent (Invitrogen). Reverse transcription with 200 ng of cDNA was performed with the High Capacity cDNA Reverse Transcription Kit (Applied Biosystems) in accordance with the manufacturer’s recommendation. Real-time PCR was performed on 7300 Real-Time PCR System (Life Technologies Applied Biosystems, Grand Island, NY) using the following cycling profile: an initial incubation of 50°C for 2 min, 95°C for 10 min, 40 cycles at 95°C for 15 s and 60°C for 60 s. All primers were purchased from Sigma. The relative expression was calculated using the 2(–Delta Delta C(T)), as previously described (Lim et al., 2011).

### Western Blot

Temozolomide resistant GBM cells were prepared as previously described (Munoz et al., 2014b). Briefly, GBM cells were incubated with 200 μM of TMZ or vehicle (control). At different times during treatment with TMZ, whole cell extracts were isolated with M-PER Mammalian Protein Extraction Reagent (Thermo Fisher Scientific, Danvers, MA). The cell extracts (10 μg) were analyzed by western blots, as previously described (Park et al., 2013). Briefly, the samples were electrophoresed on 12% SDS-PAGE gels (Bio-Rad, Hercules, CA). The proteins were transferred onto PVDF membranes (Perkin Elmer, Boston, MA). The membranes were incubated overnight with primary antibodies at a final dilution of 1/1000. Primary antibodies were detected during a 2-h incubation period with HRP-conjugated IgG at 1/2000 final dilution. HRP activity was detected by chemiluminescence using SuperSignal West Femto Maximum Sensitivity Substrate (Thermo Fisher Scientific, Danvers, MA). Membranes were stripped with Restore Stripping Buffer (Thermo Fisher Scientific) prior to being reprobed with other antibodies.

### Short Interference RNA (siRNA) Targeted Knockdown

Dicer1 targeting-siRNA pooled duplexes (Dharmacon, Lafayette, CO) knocked down Dicer1 in GBM cells using methods, previously described (Munoz et al., 2014c). Briefly, GBMs (10^4^) were seeded in 12-well plates. After 24 h, 30 nM siRNA was delivered into the cells using Lipofectamine RNAiMax (Life Technologies). Control cells were transfected from non-targeting siRNA duplexes (Dharmacon).

### miRNA Analyses

Stem Cell miRNA Profiling Panel with 95 miRNAs were purchased from System Biosciences (Palo Alto, CA). The analyses followed manufacturer’s protocol. Total RNA was isolated from exosomes using Trizol^®^ and then analyzed by qPCR. Since nuclear U6 internal control would not be detected in exosomes we selected the miRNAs with absolute changes between vehicle and TMZ-resistant cells. In this regard, the selected miRs were undetectable in vehicle-treated cells and detectable in with TMZ resistance.

### Cell Cycle Analyses

Cell cycle analyses were performed by labeling with propidium iodide (PI) or 7-AAD, as described (Lim et al., 2011). GBM cells (10^6^) were washed in PBS and then resuspended in 0.1% hypotonic sodium citrate solution containing 5 μg/ml PI and 200 μg/ml DNase-free RNase A. Cells were incubated for 30 min at room temperature and then immediately acquired on FACSCalibur (Becton Dickson, San Jose, CA) and then analyzed with the ModFit software (Verity Software House). 7-AAD labeling were done with 1 μg/mL for 20 min. After this, the cells were washed and then immediately analyzed as for PI labeling.

### Caspase Activity

GBM cells (10^4^) were studied for caspase activity using Vybrant^®^ FAM Caspase-3 and -7 Assay Kit (Thermo Fisher Scientific). The method was performed according to the manufacturer’s protocol. The cells were evaluated by flow cytometry using the FACS Calibur (Becton Dickinson).

### Exosome Isolation and Characterization

Exosomes were isolated using a multi-step process. First, large particles were removed by sequential centrifugation up to 50,000 *g* (Lim et al., 2011). The remaining particles were pelleted by ultracentrifugation (Sorvall mTx 150, Thermo Fisher Scientific, Springfield, NJ) at 100,000 *g* for 18 h. The recovered vesicles were analyzed for tetraspaninins (CD63 and CD81) by western blot and flow cytometry. The latter method used CD63 magnetic bead isolation. The exosomes were captured onto the beads and then labeled with CD63-FITC and anti-CD81-APC (BD Biosciences).

The recovered particle size was verified by Nanoparticle tracking analysis (NTA) using a NanoSight NS300 instrument (Amesbury, United Kingdom) as described (Bliss et al., 2016). The data were analyzed with the NTA software (NANOSight version 2.3) using dilutions with deionized water.

### Statistical Analyses

Data were analyzed using the students *t*-test using two comparable groups (control/experimental). A *p* value of less than 0.05 was considered significant.

## Results

### Analyses of GBM Cell-Derived Exosomes

**FIGURE 1:**
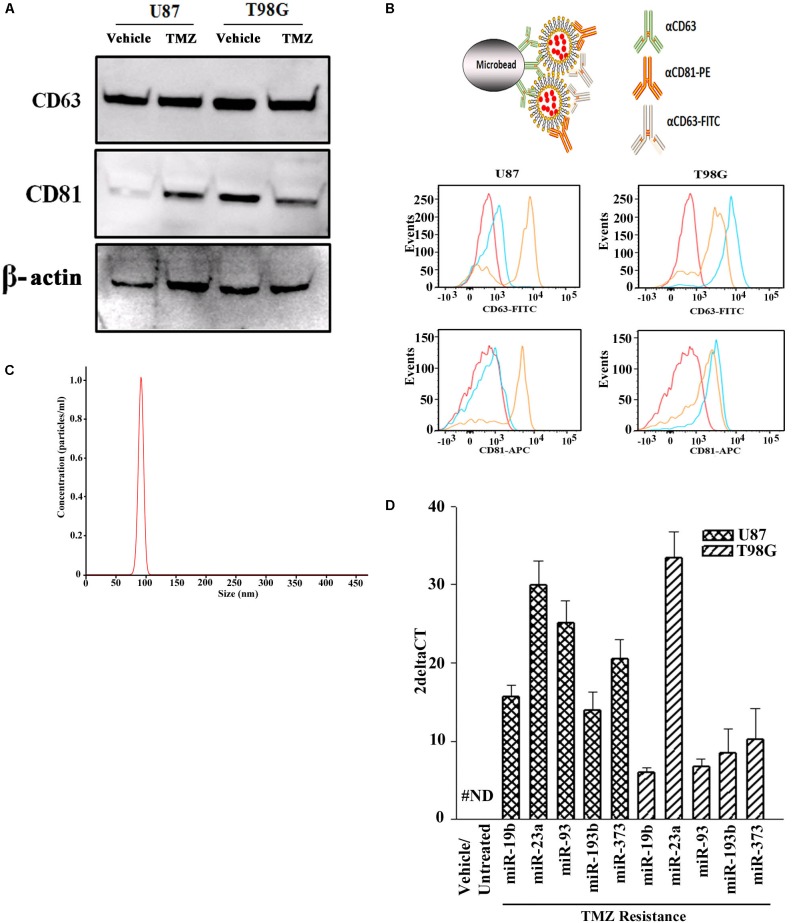
miRNA profile in TMZ resistant GBM cells (U87 and T98G). **(A)** Exosomes were collected from vehicle- and TMZ-treated GBM cells and then analyzed for CD63 and CD81 by western blot. The membrane was stripped and reprobed for β-actin. **(B)** The carton (top) demonstrates how exosomes were immunoprecipitated with microbeads conjugated to anti-CD63. The microbeads were incubated with exosomes from vehicle- or TMZ-resistant GBM cells. After this, the beads were incubated with anti-CD81-PE and anti-CD63-FITC. Control beads were incubated with isotype control. The beads were analyzed by flow cytometry: red, negative/isotype control, blue untreated, yellow TMZ-treated). **(C)** Additional analyses of the exosomes were done by NTA. A represented histogram is shown demonstrating the average size of 100 nm. **(D)** The miRNAs from the arrays in TMZ-resistant cells and naïve (untreated and vehicle treatment) GBM cells. The results are presented as 2^Δ^CT (*n* = 3, ±SD).

Prior to testing the role for exosome-containing miRNA in TMZ-resistance, we studied the exosomes by phenotype and size to ensure no contamination with other microvesicles such as apoptotic bodies. Exosomes were isolated from the culture media of GBM cells, treated with vehicle (DMSO) or with TMZ (induced resistant cells). The latter was achieved with 200 μM TMZ for 72 h, as described (Munoz et al., 2014a). Due to the endosomal origin of exosomes, they were characterized for two tetraspanin proteins, CD63 and CD81. Western blot showed bands for CD63 and CD81 with a relatively light band for vehicle-treated U87-derived exosomes (Figure 1A). A second set of analyses used metallic microbeads with bound anti-CD63 to capture all exosomes (Figure 1B, top). The exosomes were detected by double labeling with anti-CD63-FITC and anti-CD81-APC. Flow cytometric analyses indicated expressions of CD63 and CD81, although with varied fluorescence intensities (Figure 1B, lower panels). The size of exosomes were analyzed by NTA, which showed a narrow histogram with average size of 100 nm, indicating homogeneity of the exosome size (Figure 1C; Beach et al., 2014).

### Selected miRNAs in TMZ-Resistant Exosomes

Next, we asked if the contents of exosomes might begin to explain the cyclin state of GBM resistance. We compared the exosomal miRNAs from TMZ-resistant U87 and T98G cells with vehicle (DMSO)- treatment using a PCR-based array with 95 miRNAs linked to cell cycle. We selected those that showed an absolute increase from vehicle for each cell line. Next, we narrowed the selection for those that showed consistency in both cells lines. This resulted in five miRNAs (miR-19b, 23a, 93, 193b, and 373) (Figure 1D). The left bar was included to show that these five miRNAs were undetected in the untreated and vehicle-treated GBM cells (#ND = not detected).

**FIGURE 2:**
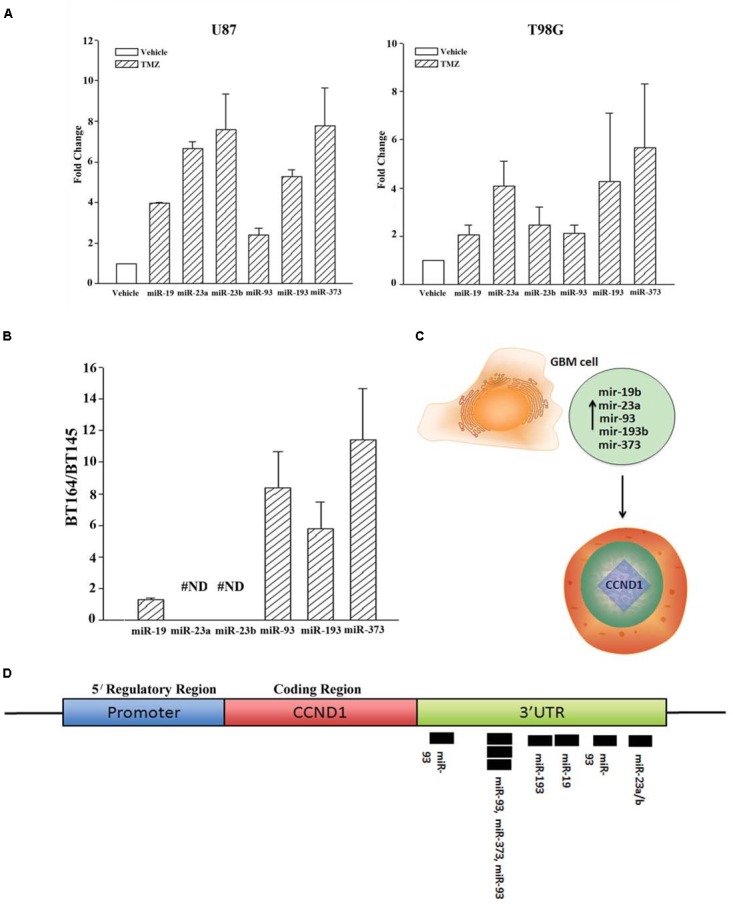
Validation of miRNAs from TMZ-resistant GBM cells and identifying the target. **(A)** Real time PCR was performed for the miRNAs shown to be increased in array studies (Figure 1D). Real-time PCR for miR-19, -23a/b, -93, -193 and -373 was performed with RNA from vehicle-treated and TMZ-resistant U87 and T98G cells. The values for vehicle were assigned 1 to calculate fold changes in the TMZ-resistant GBM cells (*n* = 4 ± SD). **(B)** Real time PCR was performed for the miRNAs studied in “A” using RNA from low-passage BT164 (TMZ resistant) and BT145 (TMZ naïve). The results are presented as the ratio of normalized Ct values of BT164/BT145 (*n* = 4, ±SD). ND = none detected. **(C)** Ingenuity^®^ miRNA analyses identified specific miRNAs as targets for *CCND1* (Cyclin D1). **(D)** The miRNAs, identified in “C,” are shown for predictive binding sites on the 3′UTR of cyclin D1 (CCND1).

We next validated the array studies by real-time PCR using RNA from naïve (untreated and vehicle treatment) and TMZ-resistant U87 and T98G cells. The resistant cells were acquired by treating with 200 μM TMZ for 72 h. In addition to the five miRNAs shown in Figure 2A, we also included miR23b. The values obtained with exosomes from vehicle and untreated GBM cells were similar and were arbitrarily assigned values of 1. The changes in miRNAs from TMZ-resistant exosomes were presented as fold change over vehicle/untreated exosomes. MiR-19b, 23a/b, 93, 193b, and 373 expressions ranged between 2 and 8 folds (Figure 2A). Based on these results, we experimentally assigned these miRNAs as the signature profile for TMZ-resistant GBM cells.

### Narrowing the Signature miRNA Profile in Early Passage Primary GBM Cells

U87 and T98G cells were long-term passaged cells that were artificially established as chemoresistance GBM cells. We therefore asked whether the identified profile of miRNA was similar to low-passage GBM cells from patients. We used two primary cells that were established as neurospheres from GBM patients. These cell lines were previously described (Munoz et al., 2014a). BT145 was derived from a patient that never received TMZ therapy and BT164, from a patient refractory to TMZ treatment. Both cell lines retained similar properties in culture with regard to TMZ sensitivity.

RNA from neurosphere cultures (BT145 and BT164) were analyzed by TaqMan^®^ qPCR for miR-19b, 23a/b, 93, 193b, and 373. The results showed >2 fold increases in miR-93b, 193 and 373 in BT164 cells (TMZ resistant) as compared to BT145 cells (TMZ sensitive) (Figure 2B). MiR-19 was <2 fold in BT164/BT145 cells. MiR-23a and MiR-23b were undetected in both cell lines. Based on these results, further analyses on TMZ resistance focused on miR-93b, 193 and 373.

### Predicted Function for miR-93b, 193 and 373

We used Ingenuity^®^ miRNA target analyses from reports in the literature and identified miR-19, 23a/b, 93b, 193 and 373 as individual target of CCND1 (Cyclin D1) (Figure 2C; Supplementary Material). We therefore mapped these miRNAs on the CCND1 3′ UTR of Cyclin D1 (*CCND1*) using TargetScan^®^. The analyses revealed a cluster of miR-93 and miR-193 in the 3′ UTR of CCND1 (Figure 2D). We next examined previous information from >500 Agilent arrays from The Cancer Genome Atlas (TCGA) and also noted increases in miR-93b and miR-193 (Munoz et al., 2014a). Based on these we narrowed the number of miRNAs to -93 and -193 in further studies.

### Decreased Cyclin D1 in TMZ-Resistant GBM Cells

**FIGURE 3:**
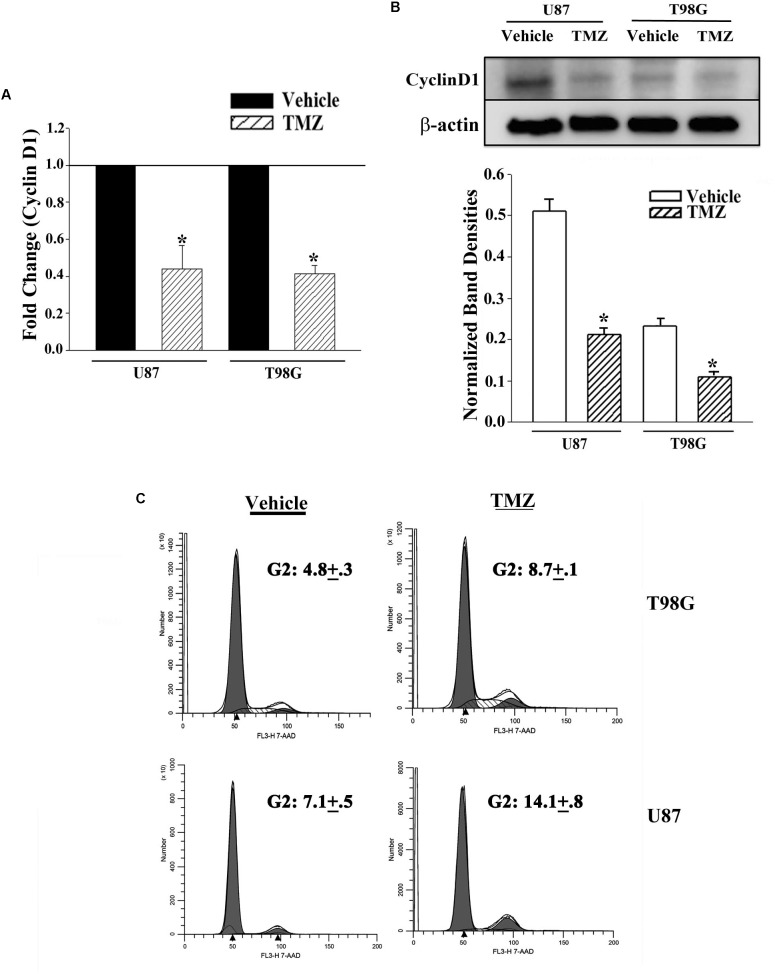
Decrease in Cyclin D1 in TMZ resistant GBM cells and cycling quiescence. **(A)** Real time PCR was performed with RNA from vehicle- and TMZ-treated U87 and T98G for Cyclin D1 mRNA. The normalized values for vehicle treatment are assigned 1 and the treated cells are presented as fold change (±SD, *n* = 4). **(B)** Western blots were performed with cell extracts from vehicle and TMZ-treated U87 and T98G for Cyclin D1. The blot was stripped and reprobed for β-actin. The normalized band densities are presented as the mean ± SD of three different experiments. **(C)** U87 and T98G cells were labeled with 7-AAD in three independent experiments. The figure shows representative histograms. ^∗^ *p* < 0.05 vs. vehicle treatment.

The results shown in Figure 2 predicted miR-23, -93 and 193 as targets of Cyclin D1. However, we did not pursue studies on miR-23 since it was not detected in primary resistant GBM spheres. First, we studied the expression of Cyclin D1 in vehicle and TMZ-resistant GBM cells by real-time PCR and western blot. Real time PCR showed a significant (*p* < 0.05) reduction in Cyclin D1 mRNA in TMZ-treated cells (Figure 3A). Western blots also showed a significant (*p* < 0.05) reduction in Cyclin D1 in the TMZ-resistant GBM cells as compared to untreated/naïve GBM cells (Figure 3B, normalized densities in lower panel). In summary, the results showed decreased expression of Cyclin D1 in TMZ-resistant GBM cells.

### Cell Cycle Analyses of Naïve and TMZ-Resistant GBM Cells

The decrease in Cyclin D1 at the levels of mRNA and protein did not indicate function. We therefore performed cell cycle analyses using Propidium Iodide (PI) DNA labeling. Vehicle-treated U87 showed 57.3% cycling (G2+S+M), which was reduced to 14.4% in the TMZ-resistant cells (Figure 3C). Vehicle-treated T98G cells showed 48.3% cycling cells (G2+S+M), which was reduced to 32.9% in the TMZ-resistant cells. Since TMZ generally resulted in cells in G2 phase of the cell cycle, we repeated the cell cycle analyses with 7-AAD labeling. In three independent experiments, the percentages of cells in G2 phase of the cell cycle after TMZ treatment doubled as compared to vehicle (Figure 3C). In summary, the results indicated a correlation between TMZ resistant with reduced expression of Cyclin D1.

### Dicer1 Knockdown in TMZ Resistant GBM Cells Prevented a Decrease in Cyclin D1

The studies shown in Figure 3 did not prove that the miRNAs were involved in the reduction of Cyclin D or even cycling quiescence. Prior to studies on the specific miRNAs, we first asked if miRNAs are involved in cycling quiescence of TMZ resistant GBM cells. This was addressed by knockdown of Dicer, which is a type III RNase and a key enzyme in the maturation of miRNAs (Adams and Eischen, 2014; van den Beucken et al., 2014). We established resistant GBM cells in which Dicer 1 was knocked down, as previously described (Munoz et al., 2014a). U87 and T98G cells were transfected with control oligonucleotides or Dicer-targeting siRNA and then treated with 200 μM TMZ for 72 h.

**FIGURE 4:**
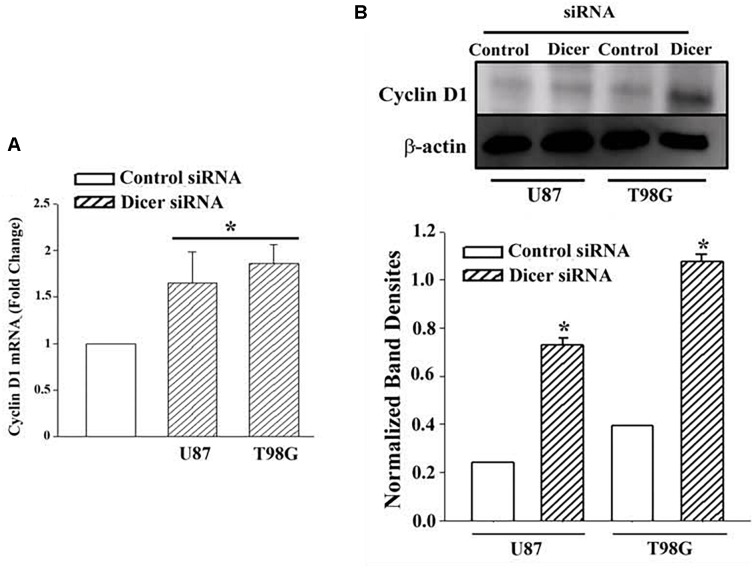
Cyclin D1 in TMZ-resistant, Dicer knockdown GBM cells. U87 and T98G cells were transfected with Dicer siRNA or control siRNA. After this, the cells were treated with 200 μM TMZ for 72 h and then analyzed for Cyclin D1 mRNA by real time PCR **(A)** and western blot **(B)**. Normalized band densities for three western blots (±SD) are shown in the graph of “A.” ^∗^*p* < 0.05 vs. control siRNA.

Real time PCR for cyclin D1 indicated ∼2-fold increase in the Dicer1 knockdown/TMZ-resistant U87 and T98G cells over control siRNA (Figure 4A). The values for control Dicer siRNA were normalized to 1 (Figure 4A). Western blot for Cyclin D1 indicated a significant (*p* < 0.05) increase (∼3 fold) in band densities in the knockdown/TMZ-resistant GBM cells, as compared to control siRNA (Figure 4B). Since dicer is needed for maturation of pre-miRs, its knockdown supported a role for miRNA in controlling the expression/decease of Cyclin D1 in TMZ-resistant GBM cells.

### Pre-miR-93 and -193 Reduced Cyclin D1 in GBM Cells

MiR-93 and -193 were increased in exosomes from TMZ-resistant cell lines and in resistant GBM spheres from a patient (short-term passage cells) (Figure 2). Furthermore, miR-93 and -193 were also increased in primary GBM tissues, based on the data deposited in TCGA [13]. Since TMZ led to a decrease in cycling cells (Figure 3C), and Dicer knockdown caused an increase in the levels of Cyclin D1 (Figure 4), we asked if miR-93 and -193 could decrease the expression of cyclin D1 in U87 and T98G cells.

**FIGURE 5:**
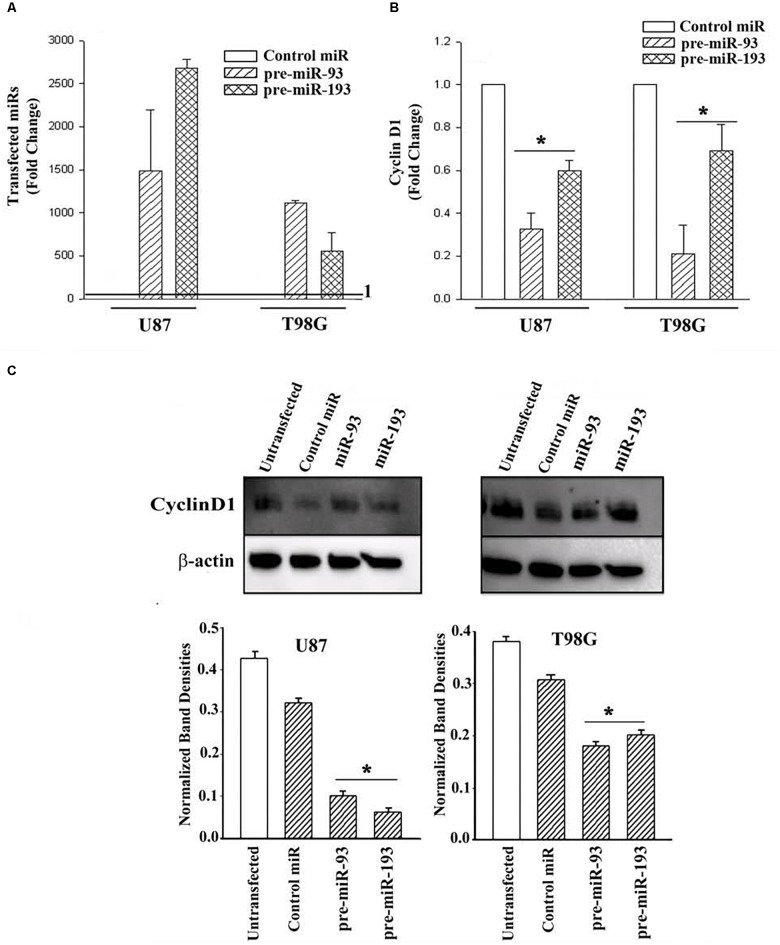
Cyclin D1 expression in miR-93/-193 GBM transfectants treated with TMZ. **(A)** Real time PCR for control miR, miR-93 and miR193 with RNA from U87 and T98G, transfected with the respective pre-miRs. The results are presented as the mean (±SD, *n* = 3) fold change of control. The controls for both primers are set at values of 1. **(B)** The transfectants in “A” were studied for cyclin D1 and the results are presented as mean fold change (±SD, *n* = 3) over the transfectants with control pre-miR. The control pre-miR was assigned values of 1. **(C)** The transfectants in “A” were treated with 200 μM TMZ. After 72 h, the viable cells were studied for Cyclin D1. An additional control with untransfected cells was included in the blot. The mean (±SD, *n* = 3) normalized band densities are shown in the graphs. ^∗^ *p* < 0.05 vs. control miR.

U87 and T98G cells were transfected with control precursor (pre)-miR, pre-miR-93 or pre-miR-193. Real-time PCR validated the efficiency of transfection, as indicated by >500-fold increase of the transfected pre-miRNAs over control pre-miRs, whose values were assigned 1 (Figure 5A). Real time PCR for Cyclin D1 indicated a significant (*p* < 0.05) decrease in Cyclin D1 mRNA in the pre-miR-93 and pre-miR-193 transfectants (Figure 5B).

Next, we investigated if pre-miR-93 and pre-miR-193 can decrease Cyclin D1 protein in the TMZ-treated GBM cells by western blots. U87 and T98G were transfected with pre-miR-93, pre-miR-193, control pre-miR or untransfected. After this, the cells were treated with 200 μM TMZ for 72 h. The cell extracts were then studied for Cyclin D1. As compared to untransfected and cells transfected with control miRNA, there was a significant (*p* < 0.05) decrease in Cyclin D1 protein in the pre-miR-93 and pre-miR-193 transfectants (Figure 5C, normalized band densities shown below for three independent experiments). In summary, this section showed pre-miR-93 and -193 as mediators of cyclin D1 expression.

### MiR-93 and -193 Enhanced TMZ-Mediated Cycling Quiescence of GBM Cells

TMZ treatment increased miR-93 and -193 levels with concomitant decrease in the cycling of GBM cells (Figure 2, 3). Also, transfection with miR-93 and -193 decreased Cyclin D1 (Figure 5). We therefore asked if miR-93 and -193 can decrease GBM cell cycle, and whether this can be enhanced by TMZ.

**FIGURE 6:**
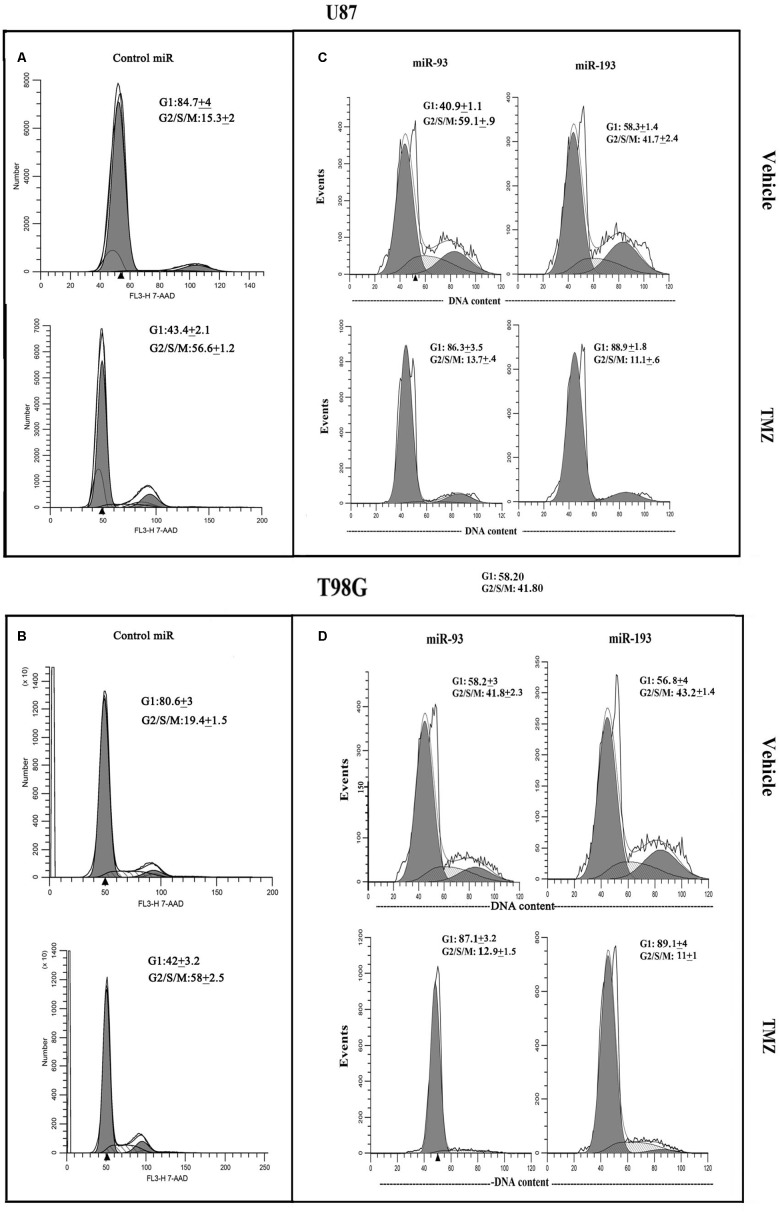
Cell cycle phase of TMZ-treated GBM cells, transfected with pre-miRs. U87 (**A** and **C**) and T98G (**B** and **D**) cells were transfected with control pre-miRs, pre-miR-93 or pre-miR-193. The cells were treated with vehicle or 200 μM TMZ. After 72 h the cells were analyzed for cell cycle by propidium iodide (miR-93 and -193) treatment and 7-AAD for control miR. The results represent three independent experiments.

U87 and T98G cells were transfected with pre-miR-93, pre-miR-193 or control pre-miRNA, and then treated with vehicle or 200 μM TMZ. At 72 h, the cells were studied for cycling phase by PI or 7-AAD labeling. Vehicle-treated U87 cells, transfected with control pre-miRNA showed 15.3% cells in cycling phase (G2+S+M), which was increased to 56.6% by TMZ, indicating arrest (Figure 6A). Transfection with pre-miR-93 led to a change in 59.1 to 13.7% cycling with TMZ (Figure 6C, left panels). Similar studies with pre-miR-193 reduced cell cycling from 41.7 to 11.1% following TMZ treatment (Figure 6C, right panels).

Studies with T98G showed similar changes with regards to cell cycle. Vehicle-treated T98G cells, transfected with control pre-miRNA, showed 19.4% cycling (G2+S+M), which was increased to 58% by TMZ treatment (Figure 6B). Vehicle-treated pre-miR-93 transfectants resulted in 41.8% cycling cells and this was reduced to 12.9% by TMZ (Figure 6D, left panels). Similarly, pre-miR-193 with vehicle showed 43.2% and decrease to 11% cycling in TMZ-treated cells (Figure 6D, right panels). In summary, in the presence of TMZ, miR-93 and -193 independently enhanced cell cycle quiescence of GBM cells.

### MiR-93 and miR-193 Induced Chemoresistance in GBM Cells

Thus far, we showed miR-93 and -193 could enhance cell cycle quiescence in GBM cells and this correlated with a decrease in Cyclin D1 (Figure 5, 6). In this set of studies, we asked if the increases in miR-93 and -193 could contribute to TMZ resistance. To address this question, we studied the cells for total and cleaved caspase 3. U87 and T98G were untransfected or transfected with control pre-miR, miR-93 or miR-193. The cells were treated with 200 μM TMZ for 72 h or with vehicle. Cell lysates were analyzed for total and cleaved caspase 3 by western blot. The percent caspase 3 activity was calculated as the band densities of cleaved caspase 3/(cleaved + total caspase 3).

**FIGURE 7:**
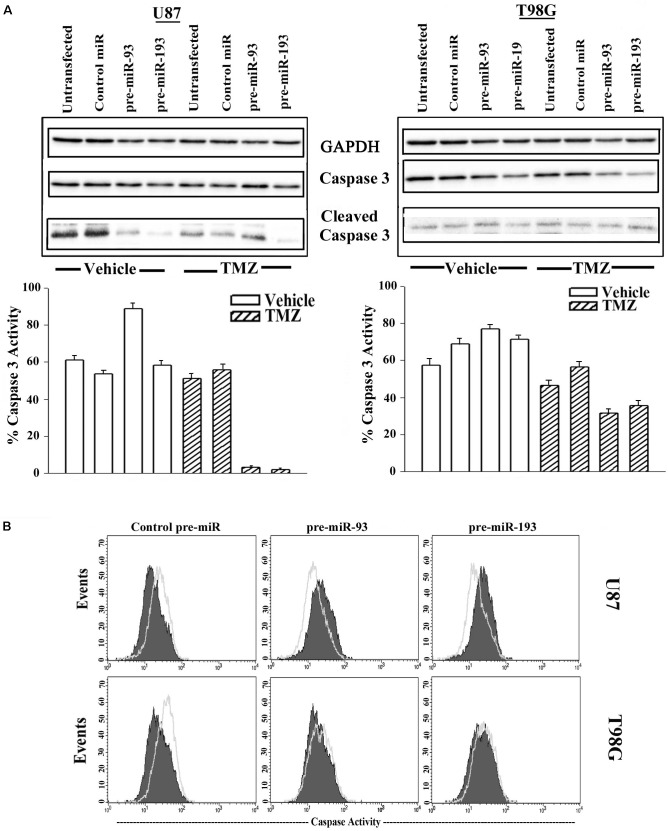
Active caspase 3 in TMZ and pre-miR-93 and -193 transfected GBM cells. **(A)** U87 and T98G cells were untransfected for transfected with control pre-miR, pre-miR-93 or -193. The cells were treated with vehicle or 200 μM TMZ for 72 h. Whole cell extracts were analyzed in western blots for total caspase 3 and cleaved caspase 3. The normalized band densities were presented as the % caspase activity ((cleaved caspase/total + cleaved caspase) × 100%) (lower graphs). **(B)** U87 and T98G were transfected with control pre-miR, pre-miR-93 or -193. The cells were treated with vehicle or 200 M TMZ for 72 h. The cells were labeled intracellularly for cleaved caspase 3 (open histogram) and total caspase (solid histogram).

Treatment of untransfected GBM cells with TMZ showed intense caspase activity and this was comparable to control pre-miR (Figure 7A, two left diagonal bars). Parallel studies with pre-miR-93 and -193 transfectants showed marked decrease in band densities (Figure 7A, two right diagonal bars). This decrease was not noted for vehicle-treated cells (Figure 7B, open bars).

Next, caspase 3 activity was studied by fluorescent DEVD (Caspase cleavage sequence). The analyses used the Vybrant^®^ FAM^TM^ caspase-3 and -7 assay. GBM cells were transfected with control pre-miR, pre-miR-93 or pre-miR-193 and then treated with 200 μM TMZ. After 72 h, the cells were analyzed by flow cytometry. The results showed reduced caspase 3 activity in U87 and T98G cells, transfected with pre-miR-93 and -193 and treated with TMZ (green histogram) as compared to vehicle (solid purple histogram) (Figure 7B). Taken together, the two approaches used to study caspase activity in GBM cells transfected with miR-93 and miR-193 indicated induced TMZ resistance.

## Discussion

GBM has continued to present with a poor prognosis for patients despite surgical intervention, chemo- and radiotherapy. Resistance to chemotherapy occurs uniformly and the mechanisms underlying this resistance are an area of extensive research (Spinelli et al., 2012). Yet, no therapies circumventing TMZ resistance have progressed to clinical therapy and improved patient outcome. This may be partially due to the diverse and numerous mechanisms employed by GBM cells to resist TMZ therapy. We have previously described three mechanisms of resistance in GBM cells treated with TMZ, increased intracellular communication (Munoz et al., 2014a), miRNA regulation of Sonic Hedgehog signaling (Munoz et al., 2014a), and EGFR regulation of the MDR1 gene (Munoz et al., 2014a). This study analyzed the changes in the miRNA content of exosomes from TMZ resistant GBM cells and then determine how this regulated Cyclin D1. These findings reveal yet another mechanism by which GBM cells resist TMZ, similar to another report (Chen et al., 2016).

Recently exosomes have become an important area of research (Beach et al., 2014). Exosomes serve as a functional method of contact-independent communication between cells and can function as a tool for cellular-based therapy (Munoz et al., 2013). Thus, we isolated exosomal miRNA from vehicle and TMZ- treated GBM cells. A miRNA array was then performed and validated with both whole cell RNA samples and neurosphere lines derived from TMZ-sensitive and –resistant patient samples (Figure 1). At that time four miRNAs were identified as solely present in TMZ resistant cells (Figure 2). When compared to data from TCGA, miR-93 and -193 were upregulated in >500 TCGA samples. Interestingly, miR-93 has been shown to regulate AKT signaling in Cisplatin-resistant Ovarian Cancer cells (Fu et al., 2012) and miR-193 has been shown to be upregulated in TMZ- resistant GBM cells, but no target has been identified (Hiddingh et al., 2014). Also, similar to our findings, miR-93 have been shown to promote the malignancy of GBM partly via TMZ resistance (Chen et al., 2016). This was an interesting report as it confirms our findings shown a link between miR-93 and Cyclin D.

miRNA Pathway analyses with Ingenuity^®^ revealed published data showing regulation of Cyclin D1 by miR-93 and miR-193 (Chen et al., 2010; Yu et al., 2011). We then looked at expression of Cyclin D1 RNA and protein in TMZ treated GBM cells and noted a significant (*p* < 0.05) reduction in its expression as compared to vehicle (DMSO)-treated cells (Figure 3A,B). Cell cycle analyses by flow cytometry also showed retention of the cells in G1/0 phase following TMZ exposure (Figure 3C). miRNA biosynthesis requires cleavage by the RNase type III, Dicer1. To evaluate a role for miRNA regulation of Cyclin D1, Dicer1 was knocked down by targeting siRNA. Dicer1 knockdown restored Cyclin D1 expression in TMZ treated cells, indicating a role for miRNA in the cycling quiescence of TMZ-treated GBM cells. Dicer1 knockdown has also been shown to increase GBM sensitivity to TMZ (Munoz et al., 2014a). We then transfected GBM cells with precursor miRNA-93 and -193 and this caused resulted in a significant (*p* < 0.05) decrease in Cyclin D1 with concomitant cycling quiescence (Figure 5C, 6). These two miRNAs also protected the GBM cells from cell death, despite the presence of TMZ (Figure 7A). Taken together, these experiments showed a role for miRNA in the regulation of Cyclin D1 in TMZ-exposed GBM cells.

miRNAs have become an area of research with great potential. miRNA not only regulate important cellular processes, but also offer an avenue of therapeutic intervention (Shibata et al., 2015). This study shows how two miRNAs, working in concert, to target Cyclin D1 to induce cycling quiescence of GBM cells. Cellular proliferation is a hallmark trait of cancer; yet, cancers cells have shown reduction of cell cycle progression as a method to evade chemotherapies depending on DNA synthesis such as alkylating agents (Miwa et al., 2015). Thus, the findings in this paper allow for the development of miRNA targeting therapy as an option to overcome this mechanism of TMZ resistance in GBM, a malignancy known to show uniform resistant to TMZ and poor patient outcome. Non-coding RNA such as miRNA can be used in clinical application via several methods. They can be expressed in mesenchymal stem cells for delivery to the tumor as exosomes. Additionally, they can be packaged into nanoparticle or even in microvesicles. The limitation of this paper is the omission of *in vivo* studies, which will be corrected in ongoing research.

## Author Contributions

JM and PR conceptualized and designed the study. JM, NW, SG, GS, and SM involved in development of methodology. JM, NW, SG, SM, and SP acquired the data. All authors analyzed and interpreted the data, and wrote and edited the manuscript.

## Conflict of Interest Statement

The authors declare that the research was conducted in the absence of any commercial or financial relationships that could be construed as a potential conflict of interest.
